# Comparison of ^99m^Tc-sestamibi and doxorubicin to monitor inhibition of P-glycoprotein function

**DOI:** 10.1054/bjoc.2000.1621

**Published:** 2001-02

**Authors:** T Muzzammil, M J Moore, D Hedley, J R Ballinger

**Affiliations:** Departments of Pharmaceutical Sciences, University of Toronto, 610 University Avenue, Toronto, Ontario, M5G 2M9, Canada; Departments of Medical Biophysics, University of Toronto, 610 University Avenue, Toronto, Ontario, M5G 2M9, Canada; Division of Experimental Therapeutics, 610 University Avenue, Ontario Cancer Institute, Toronto, Ontario, M5G 2M9, Canada; Department of Medical Oncology, 610 University Avenue, Ontario Cancer Institute, Toronto, Ontario, M5G 2M9, Canada; Department of Nuclear Medicine, Addenbrooke's Hospital, Hills Road, Cambridge, CB2 2QQ, United Kingdom

**Keywords:** P-glycoprotein, radionuclide imaging, ^99m^Tc-sestamibi, flow cytometry, doxorubicin, MCF7 human breast carcinomaell line

## Abstract

P-glycoprotein (Pgp) overexpression is a well-recognized factor in resistance to chemotherapy. Doxorubicin flow cytometry is used to monitor Pgp function in haematological specimens and biopsies from other cancers, and radionuclide imaging with sestamibi has recently shown promise for non-invasive monitoring. In the present study the two methods were directly compared in single-cell suspensions of three variants of the human breast carcinoma cell line MCF7: sensitive MCF7/WT, doxorubicin-selected MCF7/AdrR, and *MDR1* -gene-transfected MCF7/BC19 cells with doxorubicin resistance factors of 1, 192, and 14, respectively. Accumulation of sestamibi and mean fluorescence of doxorubicin (5.5 μM) were assessed over 60 min in the presence and absence of Pgp modulators GG918 (0.01 to 0.2 μM) and PSC833 (0.05 to 2.0 μM). Accumulation curves for sestamibi and doxorubicin differed among the cell variants under control conditions, with sestamibi showing a significantly greater difference between WT and resistant cells than doxorubicin. Both GG918 and PSC833 reversed uptake deficits to WT levels for sestamibi in MCF7/BC19 cells and doxorubicin in MCF7/BC19 and MCF7/AdrR cells, but failed to show the same effect for sestamibi in MCF7/AdrR cells (∼30% of MCF7/WT level). Thus, both methods clearly distinguished sensitive from resistant MCF7 variants, with the radionuclide method showing greater sensitivity. © 2001 Cancer Research Campaign http://www.bjcancer.com


British Journal of Cancer (2001) 84(3), 367–373
© 2001 Cancer Research Campaign
doi: 10.1054/ bjoc.2000.1621, available online at http://www.idealibrary.com on IDEAL®

http://www.bjcancer.com

### Comparison of $^{99m}$Tc-sestamibi and doxorubicin to monitor inhibition of P-glycoprotein function

**T Muzzammil[1,3], MJ Moore[1,3,4], D Hedley[2,3,4] and JR Ballinger[1,3,5]**

Departments of [1]Pharmaceutical Sciences and [2]Medical Biophysics, University of Toronto, 610 University Avenue, Toronto, Ontario, M5G 2M9, Canada; [3]Division of Experimental Therapeutics and [4]Department of Medical Oncology, Ontario Cancer Institute, 610 University Avenue, Toronto, Ontario, M5G 2M9, Canada; [5]Department of Nuclear Medicine, Addenbrooke's Hospital, Hills Road, Cambridge CB2 2QQ, United Kingdom

**Summary** P-glycoprotein (Pgp) overexpression is a well-recognized factor in resistance to chemotherapy. Doxorubicin flow cytometry is used to monitor Pgp function in haematological specimens and biopsies from other cancers, and radionuclide imaging with sestamibi has recently shown promise for non-invasive monitoring. In the present study the two methods were directly compared in single-cell suspensions of three variants of the human breast carcinoma cell line MCF7: sensitive MCF7/WT, doxorubicin-selected MCF7/AdrR, and *MDR1*-gene-transfected MCF7/BC19 cells with doxorubicin resistance factors of 1, 192, and 14, respectively. Accumulation of sestamibi and mean fluorescence of doxorubicin (5.5 μM) were assessed over 60 min in the presence and absence of Pgp modulators GG918 (0.01 to 0.2 μM) and PSC833 (0.05 to 2.0 μM). Accumulation curves for sestamibi and doxorubicin differed among the cell variants under control conditions, with sestamibi showing a significantly greater difference between WT and resistant cells than doxorubicin. Both GG918 and PSC833 reversed uptake deficits to WT levels for sestamibi in MCF7/BC19 cells and doxorubicin in MCF7/BC19 and MCF7/AdrR cells, but failed to show the same effect for sestamibi in MCF7/AdrR cells (~30% of MCF7/WT level). Thus, both methods clearly distinguished sensitive from resistant MCF7 variants, with the radionuclide method showing greater sensitivity. © 2001 Cancer Research Campaign http://www.bjcancer.com

**Keywords**: P-glycoprotein; radionuclide imaging; $^{99m}$Tc-sestamibi; flow cytometry; doxorubicin; MCF7 human breast carcinoma cell line

The hallmark of the multidrug resistance (MDR) phenotype is resistance of tumour cells to a variety of structurally diverse cytotoxic drugs, mainly natural products such as anthracyclines, vinca alkaloids, and taxanes (Bradley et al, 1988). One important mechanism of MDR involves cell membrane transporters such as P-glycoprotein (Pgp) (Bradley et al, 1988) and multidrug resistance-associated protein (MRP) (Cole et al, 1992), which function as drug-efflux pumps and keep cytotoxic drugs out of cells. Gene-transfection studies have confirmed the role of Pgp and MRP in MDR (Bradley et al, 1988; Belli et al, 1990; Fairchild et al, 1990; Brueninger et al, 1995) and an association has been shown between Pgp and/or MRP overexpression and clinical refractoriness in a variety of cancers (Chan et al, 1988; Kuwazuru et al, 1990; Schuurhuis et al, 1995), though high drug-resistance factors do not necessarily reflect clinical resistance.

Pgp-mediated MDR can be reversed in vitro by a variety of compounds which inhibit Pgp function and allow greater concentrations of chemotherapeutic drugs to be achieved intracellularly (Ford and Hait, 1990). Among the first-generation Pgp modulators are cyclosporin A, verapamil, and quinidine. In early attempts at modulation of Pgp function in the clinic with these drugs, the results were generally unsuccessful. This has been attributed to poor selection of patients, inadequate concentrations of modulator reaching solid tumours, and pharmacokinetic interactions between modulators and cytotoxic drugs leading to enhanced toxicity (Tunggal et al, 1999). Identification of structural features necessary for anti-Pgp activity has allowed the design of more specific and potent second-generation modulators, such as GG918 (elacridar) (Hyafil et al, 1993), PSC833 (valspodar) (Twentyman, 1988), and LY335979 (Dantzig et al, 1996), which are undergoing clinical study.

It is often difficult to determine whether an individual patient's tumour overexpresses Pgp. One method applied clinically is flow cytometry, in which cellular fluorescence intensity serves as an indicator of accumulation of a fluorescent Pgp substrate, such as doxorubicin or rhodamine-123, which is inversely correlated with Pgp efflux function. It therefore provides a rapid estimation of the cellular dynamics of drug accumulation and efflux, making possible timely modifications in treatment regimens and monitoring drug resistance (Kessel et al, 1991). The assay is quick and requires only a small sample (10 000 to 20 000 cells) for meaningful analysis. Although this is simple in cell lines, application to cells from primary tumours presents problems of tumour cell heterogeneity, contamination with host cells, and limitations in obtaining biopsy samples. Flow cytometry has therefore been most successfully applied to haematological malignancies, lymphomas and relatively few solid tumours (Xie et al, 1995).

A newer application in studies of Pgp function is the use of radionuclide imaging. Technetium-99m-labelled sestamibi, a lipophilic cationic radiopharmaceutical approved for clinical use in myocardial perfusion imaging and breast cancer detection, has been used for a number of years for imaging tumours. The observation that variable accumulation in cell lines in vitro was due to sestamibi being a transport substrate for Pgp led to further exploration of sestamibi for application in MDR (Piwnica-Worms et al, 1993). As a transport substrate, sestamibi levels show an inverse correlation with Pgp expression (Del Vecchio et al, 1997).

*Received 18 February 2000*
*Revised 13 September 2000*
*Accepted 17 October 2000*

*Correspondence to:* JR Ballinger

Sestamibi and functional analogues, combined with various modulators of Pgp function, have been studied in vitro, in animal models, and extended to the clinic (Del Vecchio et al, 1999; Hendrikse et al, 1999).

Because of the combined potential of sestamibi imaging and doxorubicin flow cytometry to assess Pgp function, we undertook a comparison of the two methods in the same in vitro system in order to determine the relationship between their results and to assess the diagnostic and predictive value of sestamibi imaging. For the present study, a human breast cancer cell line was chosen because MDR is a common problem in breast cancer (Trock et al, 1997), sestamibi imaging has been applied in this type of cancer (Del Vecchio et al, 1997), and both drug-selected MCF7/AdrR and *MDRI*-gene-transfected MCF7/BC19 variants of the human breast cancer cell line MCF7/WT were available (Batist et al, 1986; Fairchild et al, 1990). These variants were included to separate the effects of Pgp alone (MCF7/BC19) in MDR from the collective effects of other factors (MCF7/AdrR) associated with MDR phenomena. The effects of the potent second-generation Pgp modulators GG918 and PSC833 were studied on accumulation of both agents and on doxorubicin cytotoxicity in resistant cells.

### MATERIALS AND METHODS

### Cell culture

The sensitive MCF7/WT cell line was obtained from the American Type Culture Collection (Rockville, MD). The doxorubicin-selected resistant cell line MCF7/AdrR was provided by Dr G Batist (McGill University, Montreal, QC) (Batist et al, 1986) and the *MDRI*-transfected MCF7/BC19 was developed by Dr C Fairchild (Fairchild et al, 1990) and provided by Dr W Hait (Cancer Institute of New Jersey, Piscataway, NJ). The *MDRI* gene isolated from MCF7/AdrR cells had been used to transfect MCF7/WF cells to generate the MCF7/BC19 cell line, which showed a pattern of resistance similar to MCF7/AdrR cells (Fairchild et al, 1990). The resistance to doxorubicin in MCF7/AdrR and MCF7/BC19 cells was 192-fold (Batist et al, 1986) and 14-fold (Fairchild et al, 1990) relative to MCF7/WT cells. The colchicine-resistant Chinese hamster ovary cell line CHR-C5 was provided by Dr V Ling (British Columbia Cancer Agency, Vancouver, BC) as a positive control.

Alpha minimum essential medium (α-MEM) was used for all cell lines except MCF7/BC19 which was grown in RPMI-1640. Media were supplemented with 10% fetal bovine serum and the antibiotics penicillin (100 IU ml$^{-1}$) and streptomycin (100 μg ml$^{-1}$). Cells were grown at 37˚C in a humidified atmosphere of 5% $CO_2$/95% air. Single-cell suspensions at $1 \times 10^6$ cells ml$^{-1}$ were prepared by trypsinization (0.1% Bacto trypsin, Difco Labs) and cells were counted using an electronic particle counter (ZM system, Coulter Electronics Ltd, Bedfordshire, UK).

### Reagents and chemicals

Sestamibi was prepared by addition of $^{99m}$Tc-pertechnetate to kits (Dupont Pharma, Billerica, MA) and boiling for 15 min. Labelling efficiency, determined by instant thin-layer chromatography, was always above 90%. Sestamibi was diluted to 10 MBq ml$^{-1}$ in saline prior to addition to the cell preparation. Stock solutions were prepared as follows: doxorubicin (Abbott Laboratories, North Chicago, IL) 0.74 mM in PBS, stored at –20˚C; GG918 (Glaxo-Wellcome, Research Triangle, NC) 1 mM in methanol/0.1 N hydrochloric acid (4:1), freshly diluted to the required concentration with α-MEM; PSC833 (Novartis Pharmaceuticals, East Hanover, NJ) 1 mM in absolute ethanol, freshly diluted in absolute ethanol.

The primary C219 antibody (1 μg ml$^{-1}$) was a gift from Dr V Ling (British Columbia Cancer Agency, Vancouver) and secondary anti-mouse IgG and all reagents for Western blotting were purchased from Sigma Chemicals.

### $^{99m}$Tc-sestamibi accumulation

The experimental protocol was same as reported previously by our group (Ballinger et al, 1995, 1996, 1997; Muzzammil et al, 1999). Briefly, single-cell suspensions at $1 \times 10^6$ cells ml$^{-1}$ were incubated with stirring in a 37˚C water bath for 5 min to allow equilibration. Appropriate concentrations of modulator (0.01 to 0.2 μM GG918 and 0.05 to 2.0 μM PSC833) were then added and incubated for 15 min. Sestamibi was added at a final concentration of 0.1 MBq ml$^{-1}$ and samples were taken after 0.5, 15, 30, 45 and 60 min. Duplicate aliquots of 500 μl were transferred into 1.5-ml microcentrifuge tubes containing 500 μl ice-cold PBS, centrifuged for 2 min at 14 000 ***g*** in a refrigerated microcentrifuge, and the pellets were washed carefully with ice-cold saline. The supernatant was aspirated and the tips of the tubes containing the pellets were clipped into gamma counting tubes and assayed for radioactivity in a gamma well counter along with an appropriate standard. The accumulation ratio was calculated as the ratio of activity concentration inside the cell to that outside ($C_{in}/C_{out}$) in an equal volume of supernatant medium using an independent measurement of cell volume as described previously (Ballinger et al, 1995).

### Doxorubicin flow cytometry

Cell concentrations, modulator drug concentrations, incubation times, and sampling times were exactly the same as in the above experiments. Addition of doxorubicin at a final concentration of 5.5 μM defined time zero. At each time point, samples were poured into cytometry tubes containing ice-cold PBS, kept on ice and filtered through a nylon sieve before measurements to ensure single-cell analysis. Cell-associated doxorubicin fluorescence was determined with an EPICS-XL-MCL flow cytometer (Coulter Electronics). For calibration, instrument response was checked before each batch of sample measurements by standard DNA-check beads (Coulter). Fluorescence was excited at 488 nm and emission wavelengths between 550 and 600 nm were detected with a 575-nm band pass filter. Information was collected for 10 000 cells in a gated region at 100 to 300 cells s$^{-1}$. Mean doxorubicin fluorescence associated with cells was analysed from single histograms of relative fluorescence intensity and two-dimensional contours of forward scatter and 90˚ scatter. Results were presented as mean fluorescence and autofluorescence was subtracted from each measurement before analysis.

### Western blot analysis and immunohistochemical staining

Membrane preparations made from the three MCF7 variants and CHR-C5 were immunoblotted with the mouse monoclonal primary antibody C219 directed against Pgp, followed by a horseradish-peroxidase-conjugated secondary antibody which was

© 2001 Cancer Research Campaign detected by chemiluminescence. Band densities were determined using a computing densitometer and the density-to-amount of protein relationship determined for CHR-C5 cell line was used to express the relative Pgp content in each cell line.

Cytospin slides were prepared at a concentration of $1 \times 10^6$ cells $ml^{-1}$ and stained for Pgp using the JSB-1 primary antibody.

### Cytotoxicity assays

For each cell line, $1 \times 10^5$ cells were cultured in T25 tissue culture flasks 10 to 12 h prior to addition of any drugs. Attached cells were incubated with 2 μM PSC833 for 15 min before addition of doxorubicin at 1.8 and 5.5 μM. After 1- and 4-h incubations, cells were trypsinized and seeded at 200 to 12 000 cells per 60-mm tissue culture plate, depending on doxorubicin concentration, modulator treatment, cell line variant, and incubation time. Colonies were allowed to develop for 10 to 12 days and results were determined as percent survival of colonies developing under doxorubicin/PSC833-treated conditions relative to controls.

### RESULTS

### In vitro studies of sestamibi accumulation and effects of Pgp modulators

The time course of accumulation of sestamibi in wild-type and resistant variants of MCF7 cells under control conditions is shown in Figure 1A. Cellular uptake of sestamibi was continuous and accumulation ratios of 40.5 ± 6.7 (mean ± SD) in MCF7/WT cells, 3.92 ± 0.37 in MCF7/BC19 cells, and 0.64 ± 0.02 in MCF7/AdrR cells ($n = 3$) were seen at the end of 60 min, which indicates much higher activity concentration inside cells than in the surrounding medium. The percent uptakes relative to MCF7/WT cells were therefore 9.7% in MCF7/BC19 cells and 1.6% in MCF7/AdrR cells, and the two resistant cell lines showed a 6-fold difference in their sestamibi uptake. A plateau was achieved in both resistant cell lines by 30 min while in MCF7/WT cells the rate of accumulation had decreased by 60 min but no plateau was evident. Addition of a Pgp modulator caused dose-dependent inhibition of Pgp-efflux function and increased tracer accumulation to a different extent in both resistant cell lines. In MCF7/BC19 cells, 0.01 μM GG918 and 2.0 μM PSC833 restored sestamibi accumulation to MCF7/WT levels and the shape of the accumulation curves became similar to MCF7/WT controls (Figures 2A and 2B). In MCF7/AdrR cells, the same doses of both modulators caused only partial reversal of Pgp-efflux function and restored 29% to 34% of sestamibi levels seen in MCF7/WT cells (Figures 2C and 2D). A modest effect (<20%) of modulators was seen in MCF7/WT cells. $EC_{50}$ for restoration of sestamibi accumulation was <0.05 μM for GG918 and <0.05 μM for PSC833. For PSC833, the maximum effect on Pgp reversal was seen by 30 min at all concentrations, after which there was a decline. For GG918, the effect was continuous over 60 min and its relative potency with respect to PSC833 was 10-fold.

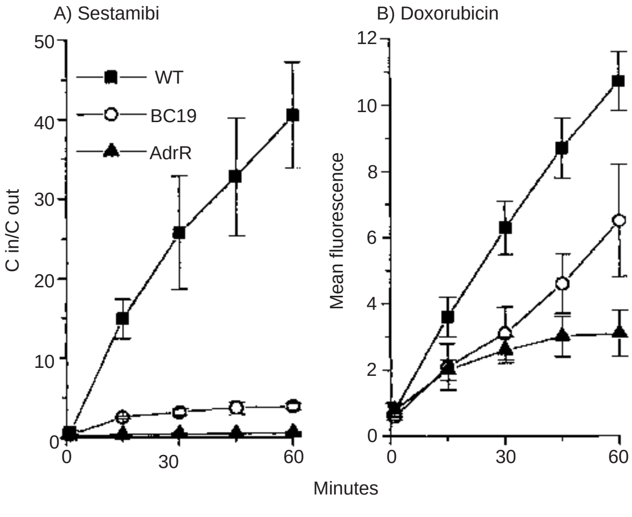

**Figure 1** Accumulation of sestamibi (panel A) and doxorubicin (panel B) in MCF7/WT, MCF7/BC19, and MCF7/AdrR cells in vitro as a function of time. Each point is the mean ± SD of 3 independent experiments. Where error bars are not shown, SD is smaller than the size of the symbol

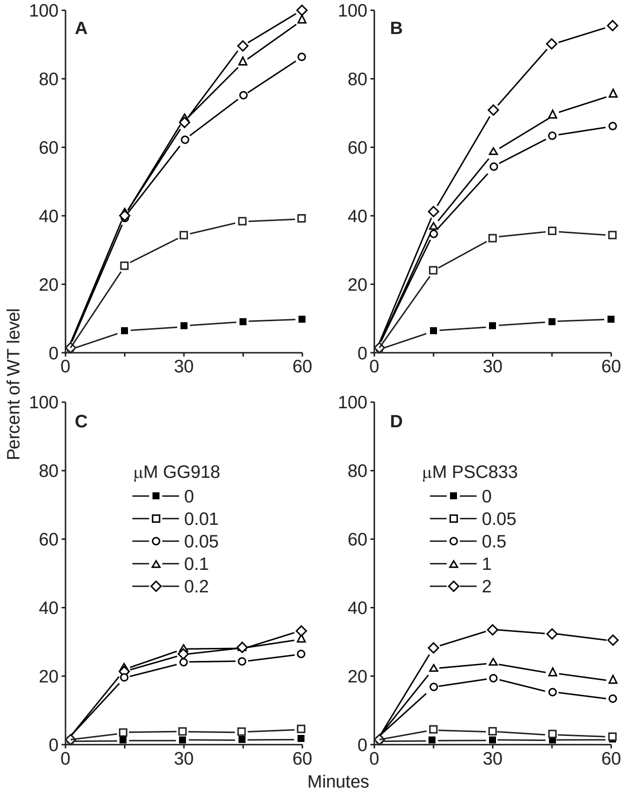

**Figure 2** Effects of addition of modulators GG918 (panels A and C) and PSC833 (panels B and D) on sestamibi accumulation in resistant variants MCF7/BC19 (panels A and B) and MCF7/AdrR (panels C and D) cells. Results are expressed as % of 60-min accumulation levels in MCF7/WT cells. Each point is the mean of 3 independent experiments and the average SD is 20% of the mean

### In vitro studies of doxorubicin accumulation and effects of Pgp modulators

The time course of accumulation of doxorubicin in wild-type and resistant cells under control conditions is shown in Figure 1B. Doxorubicin uptake in cells continued over the 60-min time course and mean fluorescence values were 10.86 ± 0.88 for MCF7/WT cells, 6.46 ± 1.66 for MCF7/BC19 cells, and 3.12 ± 0.66 for MCF7/AdrR cells ($n = 3$) at the end of incubation. Percent uptakes relative to MCF7/WT cells were therefore 59% in MCF7/BC19

© 2001 Cancer Research Campaign

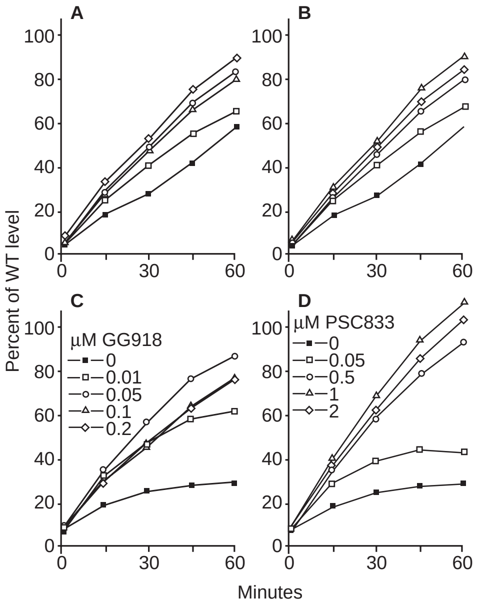

**Figure 3** Effects of addition of modulators GG918 (panels A and C) and PSC833 (panels B and D) on doxorubicin accumulation in resistant variants MCF7/BC19 (panels A and B) and MCF7/AdrR (panels C and D) cells. Results are expressed as % of 60-min accumulation levels in MCF7/WT cells. Each point is the mean of 3 independent experiments and the average SD is 37% of the mean and 29% in MCF7/AdrR cells, and the two resistant cells showed a 2-fold difference in their doxorubicin uptake. In MCF7/BC19 cells, an increase in doxorubicin uptake to MCF7/WT levels was seen with 0.2 µM GG918 and 0.1 µM PSC833 (Figures 3A and 3B), while in MCF7/AdrR cells, similar effects were seen with 0.05 µM GG918 and 1.0 µM PSC833 (Figures 3C and 3D). Modulator dose–response curves indicated $EC_{50}$ values (0.05 µM) for doxorubicin uptake which were lower than was observed for sestamibi. Unlike sestamibi, no significant effects of modulators were seen in MCF7/WT cells.

### Western blot and immunohistochemical analysis of P-glycoprotein in cells

Analysis of 1- to 20-µg membrane protein samples from the CHR-C5 cell line showed a linear relationship up to 10 µg protein with a correlation coefficient of 0.96. From this standard curve, the Pgp content was calculated for 10-µg membrane protein samples from each of the cell-line variants. The Pgp expression in MCF7/BC19 and MCF7/AdrR cells was not significantly different at 3.42 ± 1.67 and 4.06 ± 1.86 µg, respectively ($n$ = 3), and no detectable Pgp expression was observed in MCF7/WT cells. Staining for Pgp with the monoclonal antibody JSB-1 showed heterogeneous expression of this protein in MCF7/BC19 cells and a more uniform expression in MCF7/AdrR cells (data not shown). MCF7/AdrR cells, however, showed evidence of other morphological changes, as reported previously (Sommers et al, 1994).

### Cytotoxicity assay

Figure 4 demonstrates that MCF7/BC19 and MCF7/AdrR cells (open symbols) were highly resistant to doxorubicin in comparison to MCF7/WT cells, but that addition of 2 µM PSC833 (closed symbols) enhanced the cytotoxicity of doxorubicin 3- to 30-fold in the resistant cell lines. Although the results in Figure 3 show that 2 µM PSC833 results in equivalent concentrations of doxorubicin in MCF7/BC19 and MCF7/AdrR cells, Figure 4 suggests a difference in cytotoxicity of 2- to 5-fold.

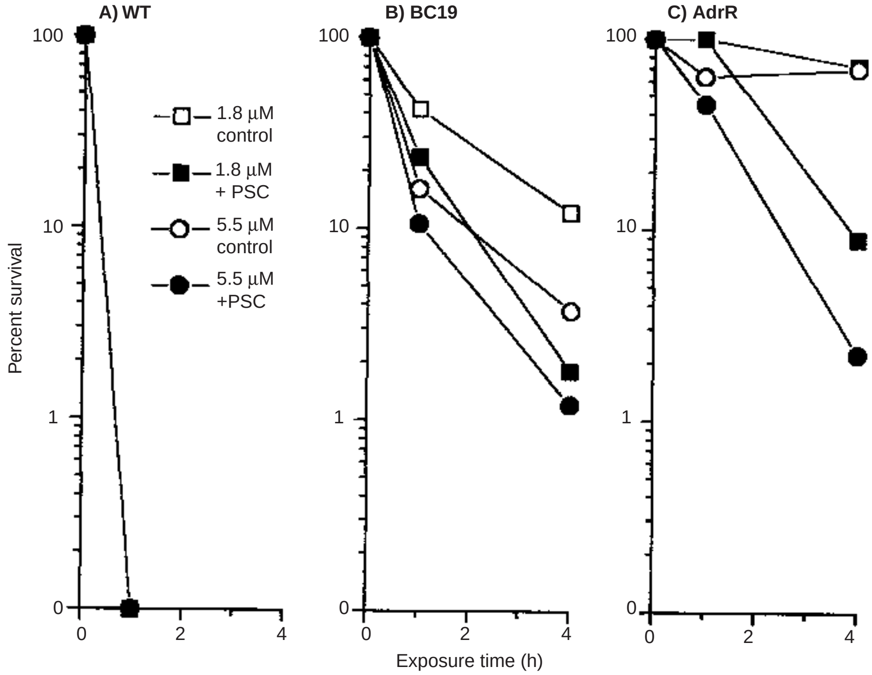

**Figure 4** Percent survival of MCF7/WT (panel A), MCF7/BC19 (panel B), and MCF7/AdrR (panel C) cells following exposure to doxorubicin (1.8 or 5.5 µM) in the presence or absence of 2 µM PSC833. Each point is the mean of 2 to 3 independent experiments

*© 2001 Cancer Research Campaign*

### DISCUSSION

Early detection of MDR in the course of therapy is important in cancer management but detection is made difficult by the multiple, diverse mechanisms of MDR, which include additional transporters and alteration in topoisomerase II and glutathione-S-transferase activity. Currently, Pgp is determined on biopsy samples and, aside from leukaemia in which tumour cells are readily available, repeat sampling is limited. Flow cytometry is a functional assay, while other in vitro techniques such as quantitative PCR and immunoblotting determine Pgp expression at molecular and protein levels, respectively. However, it is not generally possible to verify that Pgp function has been inhibited during modulator studies and non-invasive detection of transporter-mediated resistance remains an important goal.

Flow cytometry provides quantitative information on Pgp function, on chemotherapeutic drug behaviour, and on effectiveness of Pgp modulators (Kessel et al, 1991). Although these measures make it possible to monitor onset of MDR and to make timely modifications in treatment regimens, the need for biopsy samples has limited the application of flow cytometry in most solid tumours. Sestamibi imaging is a more recent approach to characterization of Pgp status in tumours and its use is expanding. This method provides a rapid, non-invasive, and repeatable assessment of cellular and tumour Pgp function and in monitoring of Pgp function modulation and response to chemotherapeutic treatment (Del Vecchio et al, 1999; Hendrikse et al, 1999).

In the present study, both doxorubicin and sestamibi shared substrate properties for Pgp and our results show their similar affinity for Pgp-transport function (Figures 2A, 2B, 3A and 3B). The differences in results for the two drugs were a function of other MDR factors that affected sestamibi uptake more than doxorubicin. This is most evident in the incomplete reversal of MDR function in the drug-selected MCF7/AdrR cell line that expresses MDR due to collective factors (Figures 2C, 2D, 3C and 3D). This study demonstrates that in cases in which MDR is due to Pgp alone, as in the MCF7/BC19 cell line, complete reversal is possible for both drugs.

In drug-selected resistant cell lines, changes have been noted in membrane, cytosolic, and nuclear proteins, drug conjugation enzymes, cell morphology, metabolic function, mitochondrial density, membrane potential charge, and sensitivity of target structures (Batist et al, 1986; Bradley et al, 1988; Cole et al, 1992; Piwnica-Worms et al, 1993; Schneider et al, 1994; Sommers et al, 1994). Because sestamibi has been reported to be a substrate for MRP as well as Pgp (Hendrikse et al, 1998), additional transporters in MCF7/AdrR cells (possibly including MRP) may have functioned as drug-efflux pumps for both drugs. MRP in MCF7/AdrR cells was not measured in the present study, but Benderra et al (2000) have recently shown that MCF7/AdrR cells exhibit moderate levels of *MRP1* gene by quantitative RT-PCR and MRP protein by flow cytometry. In addition, other MDR changes in MCF7/AdrR cells may have affected uptake and retention factors of sestamibi more than doxorubicin. In particular, changes in mitochondrial density and membrane potential could be important.

Accumulation of doxorubicin is a function of free diffusion of un-ionized drug which is extensively metabolized and retained in cells by binding to DNA and plasma membrane phospholipids and storage in intracellular compartments. Inhibition of Pgp function by modulators and extensive metabolism with large number of target sites for this drug may account for higher uptake of doxorubicin in resistant cells relative to sestamibi. In MCF7/AdrR cells, high levels of Pgp and a 45-fold increase in glutathione-*S*-transferase (GST) have been reported (Batist et al, 1986; Fairchild et al, 1990). This acquired ability of MCF7/AdrR cells to conjugate more drug and produce large number of metabolites, which can also be fluorescent (Takanashi and Bachur, 1976), could account for the high levels of fluorescence detected in these cells (Figures 3C and 3D). *MDR1*-gene-transfected MCF7/BC19 cells that expressed resistance solely due to Pgp did not exhibit this increased effect and behaved similarly to MCF7/WT cells in presence of Pgp modulators (Figures 3A and 3B).

For sestamibi, accumulation in cells is a function of free diffusion based on electrical potentials across the plasma and mitochondrial membranes, with localization in the mitochondria by electrostatic forces (Chiu et al, 1990). Davis et al (1985) studied the cellular uptake properties of the lipophilic cationic fluorescent dye rhodamine-123 and showed that carcinoma-derived cell lines accumulated more rhodamine than normal epithelial cells. They compared uptakes in the MCF7/WT cell line to a normal CV-1 epithelial cell line and showed that >85% of the difference in uptake was a result of elevated mitochondrial membrane potentials in MCF7 cells (Davis et al, 1985). Decreased uptake of sestamibi in resistant cells has also been attributed to a lower membrane potential and reduced mitochondrial density (Piwnica-Worms et al, 1993). The high membrane potential in MCF7/WT cells and lower potentials in resistant cells may together account for the greater sestamibi uptake differences between the two cell types and therefore the apparent higher sensitivity of sestamibi for detecting MDR and its modulation, relative to doxorubicin. In the present work, greater sensitivity could have been achieved by substitution of rhodamine-123 for doxorubicin (Broxterman et al, 1996). Furthermore, as a permanently charged lipophilic cation, rhodamine-123 behaviour might have been similar to sestamibi, though it has been reported that rhodamine-123 is not transported by MRP (Vergote et al, 1998). However, we felt it important to evaluate a fluorescent drug rather than to find the best in vitro probe.

The lower membrane potentials may also help to explain our inability to completely reverse drug resistance in MCF7/AdrR cells similar to MCF7/WT and MCF7/BC19 cells. Supporting evidence for this interpretation is also seen in results for MCF7/BC19 cells. When Pgp function in MCF7/BC19 cells was reversed by modulators, these cells achieved drug levels similar to MCF7/WT cells, indicating that membrane potential changes were not present in these cells. Thus Pgp alone affected their behaviour and sestamibi results were different in MCF7/AdrR cells due to other MDR factors. This result for sestamibi in MCF7/AdrR cells was also similar to results from our laboratory with other cell lines (Ballinger et al, 1995, 1997) and in other studies where high Pgp-expressing MDR cell lines have shown only partial restoration of radiolabelled drug accumulation in the presence of Pgp modulators (Piwnica-Worms et al, 1993). Western blotting showed levels of Pgp in MCF7/AdrR and MCF7/BC19 cells to be similar.

Both GG918 and PSC833 were effective in restoring MCF7/WT drug levels in the resistant cell-line variants. These potent second-generation Pgp modulators are in clinical trials and only a few studies have been published. The $EC_{50}$ of both modulators was in the μM dose range (GG918, 0.05 to 0.1 μM; PSC833, 1.0 to 2.0 μM) but GG918 was 10 to 20 times more potent than PSC833 for doxorubicin uptake and 10 times more potent for sestamibi uptake

© 2001 Cancer Research Campaign

(Figures 2 and 3). As well as the difference in potency, there was a subtle difference in the shapes of the sestamibi accumulation curves for the two modulators. With GG918 the tracer accumulation is continuous over the 60-min time-course of the experiment whereas for PSC833 there is a peak accumulation at about 30 min followed by a slight decline (Figure 2). This can be attributed to the different mechanism of action of the two modulators: GG918 is an irreversible inhibitor while PSC833 is a competitive inhibitor (Twentyman, 1988; Hyafil et al, 1993). This difference was not evident with doxorubicin (Figure 3).

Finally, the cytotoxicity study (Figure 4) showed that PSC833 at the 2 μM dose level significantly enhanced the cytotoxic effect of doxorubicin in both resistant cell lines. However, MCF7/AdrR cells remained 2- to 5-fold more resistant, despite similar levels of doxorubicin fluorescence (Figure 3); this could be due to additional metabolic mechanisms of MDR and points out a limitation of doxorubicin flow cytometry to predict sensitivity. Indeed, the sestamibi results appear to offer more predictive value, though this experiment was limited in scope and clearly much further study is required.

Vergote et al (1998) measured the efflux kinetics of sestamibi in cell lines K562 and GLC4, and their Pgp-and MRP-overexpressing resistant variants K562/ADR and GLC4/ADR, respectively. They concluded that sestamibi is transported with similar efficiencies by Pgp and by MRP but that these efficiencies are ~100 times lower than those reported for daunorubicin by the same laboratory (Marbeuf-Gueye et al, 1998). The similarity in efficiencies of efflux of sestamibi by Pgp and MRP may be coincidental, since the parameter measured is a composite of affinity, turnover number, and transporter number. The reported 100-fold difference in efficiencies of sestamibi and daunorubicin transport in the same cell lines may seem at odds with the conclusion of the present work that sestamibi is a more sensitive indicator of Pgp function than doxorubicin. However, the doxorubicin levels in the resistant cell lines in the present work may be enhanced by non-specific binding and the presence of fluorescent metabolites, which would combine to reduce the apparent difference between parental and resistant cell lines.

Cayre et al (1999) studied the effects of single concentrations of the Pgp modulators verapamil, PSC833, and S9788 on accumulation of sestamibi and $^{3}$H-daunomycin in the human nasopharyngeal carcinoma cell line KB-3-1 and its doxorubicin-resistant variant KB-A1. It was reported that only S9788 was able to reverse the daunomycin accumulation deficit in KB-A1 cells and none of the modulators enhanced sestamibi accumulation. These results are puzzling, given that Crankshaw et al (1998) were able to show complete reversal to wild-type behaviour of sestamibi in the KB-8-5 cell line, a colchicine-selected multidrug-resistant variant of KB-3-1, and may represent co-expression of additional resistance transporters. Thus, the results of Cayre et al (1999) in KB-A1 cells may be analogous to those obtained in MCF7/AdrR cells in the present work.

In summary, both sestamibi accumulation and doxorubicin flow cytometry techniques were able to detect MDR and its modulation, and clearly distinguished sensitive from resistant MCF7 variants. In MCF7/BC19 cells, Pgp-mediated efflux of sestamibi and doxorubicin was completely reversible with Pgp modulators. However, in MCF7/AdrR cells, reversal of the accumulation deficit with modulators was complete for doxorubicin but only partial for sestamibi. Sestamibi imaging, being non-invasive, repeatable, and applicable to solid tumours, may play a role in monitoring Pgp inhibition in the clinic.

### ACKNOWLEDGEMENTS

We thank Dr G Batist, McGill University, for providing the MCF7/AdrR cell line, Dr C Fairchild, National Cancer Institute, for the MCF7/BC19 cell line; Dr V Ling, British Columbia Cancer Agency, for the CHR-C5 cell line and C219 antibody; and Patricia Firby and Meli Li for assistance with tissue culture. We also thank DuPont Pharma for Sestamibi (Cardiolite®), Novartis Pharmaceuticals for PSC833 (valspodar/Amdray®), and Glaxo-Wellcome for GG918 (elacridar). Ms T Muzzammil received personal financial support from a University of Toronto Open Fellowship.

### REFERENCES

Ballinger JR, Hua HA, Berry BW, Firby P and Boxen I (1995) $^{99}$Tc$^{m}$-Sestamibi as an agent for imaging P-glycoprotein-mediated multdrug resistance: in vitro and in vivo studies in rat breast tumour cell line and its doxorubicin-resistant variant. *Nucl Med Commun* **16**: 253–257

Ballinger JR, Bannerman J, Boxen I, Firby P, Hartman NG and Moore MJ (1996) Technetium-99m-tetrofosmin as a substrate for P-glycoprotein: in vitro studies in multidrug resistant breast tumor cells. *J Nucl Med* **37**: 1578–1582

Ballinger JR, Muzzammil T and Moore MJ (1997) Technetium-99m furifosmin as an agent for functional imaging of multidrug resistance in tumors. *J Nucl Med* **38**: 1915–1919

Batist G, Tulpule A, Sinha BK, Katki AG, Myers CE and Cowan KH (1986) Overexpression of a novel anionic glutathione-transferase in multidrug resistant human breast cancer cell line. *J Biol Chem* **261**: 15544–15549

Belli JA, Zhang Y and Fritz P (1990) Transfer of adriamycin resistance by fusion of Mr 170,000 P-glycoprotein to the plasma membrane of sensitive cells. *Cancer Res* **50**: 2191–2197

Benderra Z, Trussardi A, Morjani H, Villa AM, Doglia SM and Manfait M (2000) Regulation of cellular glutathione modulates nuclear accumulation of daunorubicin in human MCF7 cells overexpressing multidrug resistance associated protein. *Eur J Cancer* **36**: 428–434

Bradley G, Juranka PF and Ling V (1988) Mechanism of drug resistance. *Biochem Biophys Acta* **948**: 87–128

Broxterman HJ, Sonnenveld P, Feller N, Ossenkoppele GJ, Wahrer DCR, Eekman CA, Schoester M, Lankelma J, Pinedo HM, Lowenberg B and Schuurhuis GJ (1996) Quality control of multidrug resistance assays in adult leukemia: correlation between assays for P-glycoprotein expression and activity. *Blood* **87**: 4809–4816

Brueninger LM, Paul S, Gaughan K, Miki T, Chan A, Aaronson SA and Kruh GD (1995) Expression of multidrug resistance-associated protein in NIH/3T3 cells confers multidrug resistance associated with increased drug efflux and altered intracellular drug distribution. *Cancer Res* **55**: 5342–5347

Cayre A, Moins N, Finat-Duclos F, Maublant J and Verrelle P (1999) Comparative $^{99m}$Tc-sestamibi and $^{3}$H-daunomycin uptake in human carcinoma cells: relation to the MDR phenotype and effects of reversing agents. *J Nucl Med* **40**: 672–676

Chan HSL, Bradley G, Thorner P, Haddad G, Gallie BL and Ling V (1988) Methods in laboratory investigation: a sensitive method for immunohistochemical detection of P-glycoprotein in multidrug resistant human ovarian carcinoma cell lines. *Lab Invest* **59**: 870–875

Chiu ML, Kronauge JF and Piwnica-Worms D (1990) Effects of mitochondrial and plasma membrane potentials on accumulation of hexakis (2-methoxy isobutyl isonitrile) technetium (I) in cultured mouse fibroblasts. *J Nucl Med* **31**: 1646–1653

Cole SPC, Bhardwaj G, Gerlach JH, Mackie JE, Grant CE, Almquist KC, Stewart AJ, Kurz EU, Duncan MV and Deeley RG (1992) Overexpression of a transporter gene in a multidrug-resistant human lung cancer cell line. *Science* **258**: 1650–1654

Crankshaw CL, Marmion M, Luker GD, Vallabhaneni R, Dahlheimer J, Burleigh BD, Webb E, Deutsch KF and Piwnica-Worms D (1998) Novel technetium (III)-Q complexes for functional imaging of multidrug resistance (*MDRI*) P-glycoprotein. *J Nucl Med* **39**: 77–86

Dantzig AH, Shepard RL, Cao J, Law KL, Ehlhardt WJ, Baughman TM, Bumol TF and Starling JJ (1996) Reversal of P-glycoprotein-mediated multidrug resistance by a potent cyclopropyldibenzosuberane modulator, LY335979. *Cancer Res* **56**: 4171–4179

 © 2001 Cancer Research Campaign

Davis S, Weiss MJ, Wong JR, Lampidis TJ and Chen LB (1985) Mitochondrial and plasma membrane potentials cause unusual accumulation and retention of rhodamine 123 by human breast adenocarcinoma-derived MCF-7 cells. *J Biol Chem* **25**: 13844–13850

Del Vecchio S, Ciarmiello A, Potena MI, Carriero MV, Mainolfi C, Botti G, Thomas R, Cerra M, D'Aiuto G, Tsuruo T and Salvatore M (1997) In vivo detection of multidrug resistance (MDR-1) phenotype by technetium-99m-sestamibi scan in untreated breast cancer patients. *Eur J Nucl Med* **24**: 150–159

Del Vecchio S, Ciarmiello A and Salvatore M (1999) Clinical imaging of multidrug resistance in cancer. *Q J Nucl Med* **43**: 125–131

Fairchild CR, Moscow JA, O'Brien EE and Cowan KH (1990) Multidrug resistance in cells transfected with human gene encoding a variant of P-glycoprotein and glutathione-S-transferase-π. *Mol Pharmacol* **37**: 801–809

Ford JM and Hait WN (1990) Pharmacology of drugs that alter multidrug resistance in cancer. *Pharmacol Rev* **42**: 155–199

Hendrikse NH, Franssen EJF, van der Graaf WTA, Meijer C, Piers DA, Vaalburg W and de Vries EGE (1998) $^{99m}$Tc-sestamibi is a substrate for P-glycoprotein and the multidrug resistance-associated protein. *Br J Cancer* **77**: 353–358

Hendrikse NH, Franssen EJF, van der Graaf WTA, Vaalburg W and de Vries EGE (1999) Visualization of multidrug resistance in vivo. *Eur J Nucl Med* **26**: 283–293

Hyafil F, Vergely C, Vignaud PD and Grand-Perret T (1993) In vitro and in vivo reversal of multidrug resistance by GF 120918, an acridonecarboxamide derivative. *Cancer Res* **53**: 4595–4602

Kessel D, Beck WT, Kukuruga D and Schulz V (1991) Characterization of multidrug resistance by fluorescent dyes. *Cancer Res* **51**: 4665–4670

Kuwazuru Y, Yoshimura A, Hanada S, Utsunomiya A, Makino T, Ishibashi K, Kodama M, Iwahashi M, Arima T and Akiyama S-I (1990) Expression of the MDR transporter, P-glycoprotein, in acute leukemia cells and correlation to clinical drug resistance. *Cancer* **66**: 868–873

Marbeuf-Gueye C, Broxterman HJ, Dubru F, Priebe W and Garnier-Suillerot A (1998) Kinetics of anthracycline efflux from multidrug resistance protein-expressing cancer cells compared with P-glycoprotein-expressing cancer cells. *Mol Pharmacol* **53**: 141–147

Muzzammil T, Ballinger JR and Moore MJ (1999) $^{99}Tc^{m}$-sestamibi imaging of inhibition of the multidrug resistance transporter in a mouse xenograft model of human breast cancer. *Nucl Med Commun* **20**: 115–122

Piwnica-Worms D, Chiu ML, Budding M, Kronauge JF, Kramer RA and Croop JM (1993) Functional imaging of multidrug resistance P-glycoprotein with an organotechnetium complex. *Cancer Res* **53**: 977–984

Schneider E, Horton JK, Yang C-H, Nakagawa M and Cowan KH (1994) Multidrug resistance associated protein gene overexpression and reduced drug sensitivity to topoisomerase-II in human breast carcinoma MCF7 cell line selected for etoposide resistance. *Cancer Res* **54**: 152–158

Schuurhuis GJ, Broxterman HJ, Ossenkoppele GJ, Baak JPA, Eekman CA, Kuiper CM, Feller N, van Heijningen THM, Klumper E, Pieters R, Lankelma J and Pinedo HM (1995) Functional multidrug resistance phenotype associated with combined overexpression of Pgp/*MDR1* and *MRP* together with 1-β-D-arabinofuranosylcytosine sensitivity may predict clinical response in acute myeloid leukemia. *Clin Cancer Res* **1**: 81–93

Sommers CL, Byers SW, Thompson EW, Torri JA and Gelmann EP (1994) Differentiation state and invasiveness of human breast cancer cell lines. *Breast Cancer Res Treat* **31**: 325–335

Takanashi S and Bachur NR (1976) Adriamycin metabolism in man: evidence from urinary metabolites. *Drug Metab Dispos* **4**: 79–88

Trock BJ, Leonessa F and Clarke R (1997) Multidrug resistance in breast cancer: a meta-analysis of MDR1/gp170 expression and its possible functional significance. *J Natl Cancer Inst* **89**: 917–931

Tunggal JK, Ballinger JR and Tannock IF (1999) The influence of cell concentration in limiting the therapeutic benefit of P-glycoprotein reversal agents. *Int J Cancer* **81**: 741–747

Twentyman PR (1988) Modification of cytotoxic drug resistance by non-immunosuppressive cyclosporins. *Br J Cancer* **57**: 254–258

Vergote J, Moretti JL, de Vries EGE and Garnier-Suillerot A (1998) Comparison of the kinetics of active efflux of $^{99m}$Tc-MIBI in cells with P-glycoprotein-mediated and multidrug-resistance protein-associated multidrug-resistance phenotypes. *Eur J Biochem* **252**: 140–146

Xie X-Y, Robb D, Chow S and Hedley DW (1995) Discordant P-glycoprotein antigen expression and transport function in acute myeloid leukemia. *Leukemia* **9**: 1882–1887

© 2001 Cancer Research Campaign

## References

[PDF_00684] Ballinger J. R., Bannerman J., Boxen I., Firby P., Hartman N. G., Moore M. J. (1996). Technetium-99m-tetrofosmin as a substrate for P-glycoprotein: in vitro studies in multidrug-resistant breast tumor cells.. J Nucl Med.

[PDF_00678] Ballinger J. R., Hua H. A., Berry B. W., Firby P., Boxen I. (1995). 99Tcm-sestamibi as an agent for imaging P-glycoprotein-mediated multi-drug resistance: in vitro and in vivo studies in a rat breast tumour cell line and its doxorubicin-resistant variant.. Nucl Med Commun.

[PDF_00687] Ballinger J. R., Muzzammil T., Moore M. J. (1997). Technetium-99m-furifosmin as an agent for functional imaging of multidrug resistance in tumors.. J Nucl Med.

[PDF_00690] Batist G., Tulpule A., Sinha B. K., Katki A. G., Myers C. E., Cowan K. H. (1986). Overexpression of a novel anionic glutathione transferase in multidrug-resistant human breast cancer cells.. J Biol Chem.

[PDF_00693] Belli J. A., Zhang Y., Fritz P. (1990). Transfer of adriamycin resistance by fusion of Mr 170,000 P-glycoprotein to the plasma membrane of sensitive cells.. Cancer Res.

[PDF_00696] Benderra Z., Trussardi A., Morjani H., Villa A. M., Doglia S. M., Manfait M. (2000). Regulation of cellular glutathione modulates nuclear accumulation of daunorubicin in human MCF7 cells overexpressing multidrug resistance associated protein.. Eur J Cancer.

[PDF_00700] Bradley G., Juranka P. F., Ling V. (1988). Mechanism of multidrug resistance.. Biochim Biophys Acta.

[PDF_00707] Breuninger L. M., Paul S., Gaughan K., Miki T., Chan A., Aaronson S. A., Kruh G. D. (1995). Expression of multidrug resistance-associated protein in NIH/3T3 cells confers multidrug resistance associated with increased drug efflux and altered intracellular drug distribution.. Cancer Res.

[PDF_00702] Broxterman H. J., Sonneveld P., Feller N., Ossenkoppele G. J., Währer D. C., Eekman C. A., Schoester M., Lankelma J., Pinedo H. M., Löwenberg B. (1996). Quality control of multidrug resistance assays in adult acute leukemia: correlation between assays for P-glycoprotein expression and activity.. Blood.

[PDF_00711] Cayre A., Moins N., Finat-Duclos F., Maublant J., Verrelle P. (1999). Comparative 99mTc-sestamibi and 3H-daunomycin uptake in human carcinoma cells: relation to the MDR phenotype and effects of reversing agents.. J Nucl Med.

[PDF_00717] Chan H. S., Bradley G., Thorner P., Haddad G., Gallie B. L., Ling V. (1988). A sensitive method for immunocytochemical detection of P-glycoprotein in multidrug-resistant human ovarian carcinoma cell lines.. Lab Invest.

[PDF_00721] Chiu M. L., Kronauge J. F., Piwnica-Worms D. (1990). Effect of mitochondrial and plasma membrane potentials on accumulation of hexakis (2-methoxyisobutylisonitrile) technetium(I) in cultured mouse fibroblasts.. J Nucl Med.

[PDF_00725] Cole S. P., Bhardwaj G., Gerlach J. H., Mackie J. E., Grant C. E., Almquist K. C., Stewart A. J., Kurz E. U., Duncan A. M., Deeley R. G. (1992). Overexpression of a transporter gene in a multidrug-resistant human lung cancer cell line.. Science.

[PDF_00729] Crankshaw C. L., Marmion M., Luker G. D., Rao V., Dahlheimer J., Burleigh B. D., Webb E., Deutsch K. F., Piwnica-Worms D. (1998). Novel technetium (III)-Q complexes for functional imaging of multidrug resistance (MDR1) P-glycoprotein.. J Nucl Med.

[PDF_00733] Dantzig A. H., Shepard R. L., Cao J., Law K. L., Ehlhardt W. J., Baughman T. M., Bumol T. F., Starling J. J. (1996). Reversal of P-glycoprotein-mediated multidrug resistance by a potent cyclopropyldibenzosuberane modulator, LY335979.. Cancer Res.

[PDF_00739] Davis S., Weiss M. J., Wong J. R., Lampidis T. J., Chen L. B. (1985). Mitochondrial and plasma membrane potentials cause unusual accumulation and retention of rhodamine 123 by human breast adenocarcinoma-derived MCF-7 cells.. J Biol Chem.

[PDF_00747] Del Vecchio S., Ciarmiello A., Salvatore M. (1999). Clinical imaging of multidrug resistance in cancer.. Q J Nucl Med.

[PDF_00749] Fairchild C. R., Moscow J. A., O'Brien E. E., Cowan K. H. (1990). Multidrug resistance in cells transfected with human genes encoding a variant P-glycoprotein and glutathione S-transferase-pi.. Mol Pharmacol.

[PDF_00752] Ford J. M., Hait W. N. (1990). Pharmacology of drugs that alter multidrug resistance in cancer.. Pharmacol Rev.

[PDF_00754] Hendrikse N. H., Franssen E. J., van der Graaf W. T., Meijer C., Piers D. A., Vaalburg W., de Vries E. G. (1998). 99mTc-sestamibi is a substrate for P-glycoprotein and the multidrug resistance-associated protein.. Br J Cancer.

[PDF_00758] Hendrikse N. H., Franssen E. J., van der Graaf W. T., Vaalburg W., de Vries E. G. (1999). Visualization of multidrug resistance in vivo.. Eur J Nucl Med.

[PDF_00760] Hyafil F., Vergely C., Du Vignaud P., Grand-Perret T. (1993). In vitro and in vivo reversal of multidrug resistance by GF120918, an acridonecarboxamide derivative.. Cancer Res.

[PDF_00763] Kessel D., Beck W. T., Kukuruga D., Schulz V. (1991). Characterization of multidrug resistance by fluorescent dyes.. Cancer Res.

[PDF_00765] Kuwazuru Y., Yoshimura A., Hanada S., Utsunomiya A., Makino T., Ishibashi K., Kodama M., Iwahashi M., Arima T., Akiyama S. (1990). Expression of the multidrug transporter, P-glycoprotein, in acute leukemia cells and correlation to clinical drug resistance.. Cancer.

[PDF_00769] Marbeuf-Gueye C., Broxterman H. J., Dubru F., Priebe W., Garnier-Suillerot A. (1998). Kinetics of anthracycline efflux from multidrug resistance protein-expressing cancer cells compared with P-glycoprotein-expressing cancer cells.. Mol Pharmacol.

[PDF_00773] Muzzammil T., Ballinger J. R., Moore M. J. (1999). 99Tcm-sestamibi imaging of inhibition of the multidrug resistance transporter in a mouse xenograft model of human breast cancer.. Nucl Med Commun.

[PDF_00778] Piwnica-Worms D., Chiu M. L., Budding M., Kronauge J. F., Kramer R. A., Croop J. M. (1993). Functional imaging of multidrug-resistant P-glycoprotein with an organotechnetium complex.. Cancer Res.

[PDF_00781] Schneider E., Horton J. K., Yang C. H., Nakagawa M., Cowan K. H. (1994). Multidrug resistance-associated protein gene overexpression and reduced drug sensitivity of topoisomerase II in a human breast carcinoma MCF7 cell line selected for etoposide resistance.. Cancer Res.

[PDF_00785] Schuurhuis G. J., Broxterman H. J., Ossenkoppele G. J., Baak J. P., Eekman C. A., Kuiper C. M., Feller N., van Heijningen T. H., Klumper E., Pieters R. (1995). Functional multidrug resistance phenotype associated with combined overexpression of Pgp/MDR1 and MRP together with 1-beta-D-arabinofuranosylcytosine sensitivity may predict clinical response in acute myeloid leukemia.. Clin Cancer Res.

[PDF_00791] Sommers C. L., Byers S. W., Thompson E. W., Torri J. A., Gelmann E. P. (1994). Differentiation state and invasiveness of human breast cancer cell lines.. Breast Cancer Res Treat.

[PDF_00794] Takanashi S., Bachur N. R. (1976). Adriamycin metabolism in man. Evidence from urinary metabolites.. Drug Metab Dispos.

[PDF_00796] Trock B. J., Leonessa F., Clarke R. (1997). Multidrug resistance in breast cancer: a meta-analysis of MDR1/gp170 expression and its possible functional significance.. J Natl Cancer Inst.

[PDF_00799] Tunggal J. K., Ballinger J. R., Tannock I. F. (1999). Influence of cell concentration in limiting the therapeutic benefit of P-glycoprotein reversal agents.. Int J Cancer.

[PDF_00802] Twentyman P. R. (1988). Modification of cytotoxic drug resistance by non-immuno-suppressive cyclosporins.. Br J Cancer.

[PDF_00743] Vecchio S. D., Ciarmiello A., Potena M. I., Carriero M. V., Mainolfi C., Botti G., Thomas R., Cerra M., D'Aiuto G., Tsuruo T. (1997). In vivo detection of multidrug-resistant (MDR1) phenotype by technetium-99m sestamibi scan in untreated breast cancer patients.. Eur J Nucl Med.

[PDF_00804] Vergote J., Moretti J. L., de Vries E. G., Garnier-Suillerot A. (1998). Comparison of the kinetics of active efflux of 99mTc-MIBI in cells with P-glycoprotein-mediated and multidrug-resistance protein-associated multidrug-resistance phenotypes.. Eur J Biochem.

[PDF_00809] Xie X. Y., Robb D., Chow S., Hedley D. W. (1995). Discordant P-glycoprotein antigen expression and transport function in acute myeloid leukemia.. Leukemia.

